# Strategies for salvage therapy post CAR-T therapy failure in refractory/relapsed multiple myeloma patients

**DOI:** 10.3389/fphar.2025.1515555

**Published:** 2025-03-17

**Authors:** Chao Min, Xiong Zhong, Yue Cui, Hanfu Zhang, Qingming Wang

**Affiliations:** ^1^ Department of Hematology, 2nd Affiliated Hospital, Jiangxi Medical College, Nanchang University, Nanchang, Jiangxin, China; ^2^ Hrain Biotechnology Co. Ltd., Shanghai, China; ^3^ Jiangxi Provincial Key Laboratory of Hematological Diseases, Department of Hematology, The 2nd Affiliated Hospital, Jiangxi Medical College, Nanchang University, Nanchang, Jiangxi, China

**Keywords:** multiple myeloma, chimeric antigen receptor T-cells, cellular therapy failure, salvage therapy, cellular therapeutics

## Abstract

Over the past few decades, the landscape for multiple myeloma (MM) therapy has significantly advanced, largely due to the approval and introduction of new-generation proteasome inhibitors (PIs) and immunomodulatory drugs (IMiDs). Despite these advancements, MM remains incurable. In March 2021, the U.S. FDA approved the chimeric antigen receptor T-cell (CAR-T) therapy idecabtagene vicleucel (ide-cel) for relapsed/refractory multiple myeloma (R/R MM), heralding the advent of cellular therapies for R/R MM. However, due to factors such as the downregulation or loss of tumor antigen expression, T-cell exhaustion, and the influence of the tumor immune microenvironment, most R/R MM patients inevitably experience relapse following CAR-T cell therapy. Consequently, salvage therapy in the post-CAR-T setting has emerged as a critical area of research. This review discusses the potential factors leading to CAR-T therapy failure in R/R MM patients and discusses subsequent salvage therapeutic strategies, offering recommendations for addressing treatment failure in this context.

## Graphical Abstract

**Figure:**
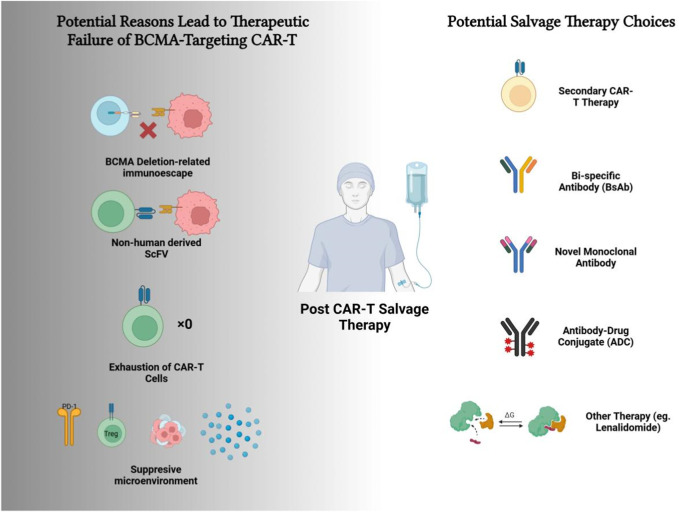
Multiple factors could result in the therapeutic failure of BCMA CAR-T therapy, while various choices for the salvage therapy could be applied.

## 1 Introduction

Multiple myeloma (MM) is a malignant proliferative disorder of plasma cells and represents the second most common hematologic malignancy. It is estimated that in 2022, 33,463 new cases of MM in the United States were diagnosed and 22,450 in China (Xia et al., 2022), respectively. The NCCN 2024 V2 guidelines recommend combination regimens as the prior-line treatment, including proteasome inhibitors (or CD38 monoclonal antibodies), bortezomib (PI), immunomodulatory drugs (IMiDs), and dexamethasone. For patients who relapse or exhibit refractory disease after one to three lines of therapy, it is advised to switch to drug combinations with different mechanisms of action. For those who have undergone four lines of treatment, novel immunotherapy such as CAR-T cell therapy or bispecific antibody (BsAb) therapy is then recommended (Kumar et al., 2023). On 5 April 2024, after rigorous evaluation, the U.S. FDA approved ciltacabtagene autoleucel (cilta-cel) for the treatment of relapsed/refractory MM (R/R MM) in patients who have previously undergone at least one line of therapy, including a proteasome inhibitor and an immunomodulatory drug, and who are refractory to lenalidomide. Consequently, cilta-cel has become the only approved BCMA-targeted therapy (encompassing CAR-T therapy, bispecific antibodies, and antibody-drug conjugates) for second-line treatment in MM patients, therefore making it regarded as one of the most promising CAR-T therapies for R/R MM. However, a meta-analysis (Hu et al., 2023) involving 761 R/R MM patients revealed that although the objective response rate (ORR) for anti-BCMA CAR-T cell therapy was a rather desirable 87%, the median progression-free survival (PFS) was only 8.77 months. Meanwhile, unfortunately, the majority of those patients demonstrated relapse within 12 months following CAR-T therapy. At present, no consensus on subsequent salvage therapy for these patients has ever reached, despite vast kinds of clinical trials have explored various approaches, including non-BCMA CAR-T cells, bispecific antibodies (BsAb), monoclonal antibodies (mAb), antibody-drug conjugates (ADC), and combination chemotherapy, with certain kinds of success (Wang et al., 2024; Keller et al., 2023; Reyes et al., 2022; Van Oekelen et al., 2023; Liu et al., 2024; Snyder et al., 2024; Bernabei et al., 2018; Chung et al., 2022; Jakubowiak et al., 2023; Grajales-Cruz et al., 2023; Ferreri et al., 2023). Therefore, in this review, we attempt to discuss the potential factors contributing to CAR-T therapy failure, the salvage therapeutic strategies that have been attempted, and the clinical outcomes, along with recommendations for some possible approaches to address these challenges.

## 2 Literature review

The mechanism of CAR-T cell therapy involves the binding of the CAR molecule on the surface of CAR-T cells to the corresponding antigen on the surface of tumor cells, which subsequently activates the intracellular signaling pathways in CAR-T cells, leading to the cytotoxic elimination of tumor cells. In the treatment of R/R MM, several target antigens have been identified for CAR-T cell therapy. B-cell maturation antigen (BCMA) is considered as one of the most promising targets due to its expression on mature B cells, normal and malignant plasma cells, and plasmacytoid dendritic cells, while being absent on early B cells, memory B cells, and normal hematopoietic stem cells (HSCs), which makes BCMA an ideal antigen of targeting. Additionally, G protein-coupled receptor class C group 5 member D (GPRC5D) is frequently utilized as a target antigen in MM CAR-T cell therapy that is highly expressed on primary MM cells but is restricted to the immune-privileged hair follicle regions in normal tissues. Notably, GPRC5D expression is independent of BCMA, allowing for the development of single or dual-targeted cellular therapies (Smith et al., 2019). Other antigens, such as CD138, CD38, and SLAMF7, are also being evaluated in ongoing clinical studies (Zhang X. et al., 2023). As of June 2024, regulatory agencies in major global pharmaceutical markets have approved four CAR-T products for the treatment of R/R MM: ide-cel, cilta-cel, equecabtagene autoleucel (equ-cel), and zevorcabtagene autoleucel (zevor-cel). Additionally, numerous other candidate CAR-T therapies are undergoing preclinical and clinical evaluation.

### 2.1 Current landscape of CAR-T cell therapy towards multiple myeloma

While CAR-T cell therapy has achieved remarkable success in treating R/R MM, the challenge of overcoming therapy failure remains a significant clinical hurdle. The 5-year follow-up data from the LEGEND-2 study on cilta-cel (Xu et al., 2024) reported an overall response rate (ORR) of 87.8%, with a 5-year progression-free survival (PFS) rate of 21.0%, and the longest duration of response extending up to 6.4 years. Meanwhile, in the phase I CRB-401 trial of ide-cel, with a median follow-up of 18.1 months, ORR reached 75.8%, with a median duration of response of 10.3 months and a median PFS of 8.8 months (Lin et al., 2023). Similarly, the FUMANBA-1 study of equecabtagene autoleucel (equ-cel) (Li et al., 2023), with a median follow-up of 13.8 months, demonstrated an ORR of 96%, although the median duration of response and median PFS has yet to be determined; the 12-month PFS rate was 78.8% (95% CI: 68.6–85.97). Additionally, the 3-year follow-up from the LUMMICAR-1 study of zevorcabtagene autoleucel (zevor-cel) (Fu et al., 2023) reported an ORR of 100%, with a median duration of response of 24.1 months and a median PFS of 25 months. Details on these outcomes of CAR-T cell therapies for R/R MM are provided in Table 1. Although these results can be regarded as promising, it is also important to acknowledge that there is a general tendency of approximately 10%–20% of the patients exhibiting resistance to CAR-T therapy, and many patients who demonstrated a response might relapse. For instance, in the LEGEND-2 trial, the 5-year progression-free survival (PFS) rate was only 21.0%, indicating that a significant proportion of patients experience disease progression post-CAR-T therapy. These data underscore the critical need for effective salvage strategies to address relapse, particularly in patients who have exhausted other treatment options. Research has identified several factors associated with less desirable PFS in the context of CAR-T therapy failure, including a history of extramedullary disease, prior BCMA-targeted therapy, elevated ferritin levels during lymphodepletion, plasma cell leukemia, and the presence of the t (4; 14) translocation (Hashmi et al., 2024a).

**TABLE 1:** Some clinical trial result of CAR-T therapy.

Trial code/Number	CAR-T product	CAR-T target	Median follow-up (months)	Efficacy			Safety	
				ORR	Median PFS (months)	Median OS (months)	CRS	ICANS
LEGEND-2 *(NCT03090659)*	LCAR-B38M (Xu et al., 2024)	BCMA	65.4	87.8%	18	55.8	-	-
CRB401 *(NCT02658929)*	bb2121 (Lin et al., 2023)	BCMA	18.1 (1.5–44.5)	75.8%	8.8 (95% CI, 5.9–11.9)	34.2 (95% CI, 19.2-NE)	75.8%	-
FUMANBA-1 *(NCT05066646)*	CT103A (Li et al., 2023)	BCMA	13.8 (0.4–27.2)	96%	NR	-	-	-
LUMMICAR-1 *(NCT03975907)*	CT053 (Fu et al., 2023)	BCMA	37.7 (14.8–44.2)	100%	25	NR	92.9%	0
POLARIS *(NCT05016778)*	OriCAR-017 (Huang et al., 2024)	GPRC5D	>24 (all patients)	100%	11.37 (95% CI, 5.93–18.00)	NR	100%	0
ChiCTR2100048888	anti-GPRC5D CAR-T (Xia et al., 2023)	GPRC5D	5.2 (3.2–8.9)	91%	NR	NR	76%	6%
NCT04662099	CS1-BCMA CAR-T (Li et al., 2024)	CS1 and BCMA	246 days (55–547)	81%	9.0 (95% CI, 2.1-NR, estimated)	NR	38%	0
NCT04236011 and NCT04182581	GC012F (Du et al., 2023)	BCMA and CD19	30.7 (14.6–43.6)	93.1%	38.0 (95% CI, 11.8-NE, estimated)	-	86.2%	0

IQR, interquartile range; NE, not evaluable.

Notably, there are significant differences in baseline characteristics among patients enrolled in different clinical trials. For example, in the LEGEND-2 study (NCT03090659), the median number of prior treatment lines was 3 (range: 2–4), whereas, in the CRB-401 trial (NCT02658929), the median number of prior treatment lines was 6 (range: 3–18). Additionally, only 10% of patients in the LEGEND-2 study had extramedullary disease (EMD), compared to 30% in the CRB-401 trial. These disparities may limit the direct comparability of efficacy outcomes [e.g., overall response rate (ORR) and progression-free survival (PFS)] across studies. Therefore, all the comparisons and contrasts across studies can only be regarded as preliminary with biases. Moving forward, standardized enrollment criteria and subgroup analyses will be essential to further elucidate the relationship between CAR-T efficacy and patient baseline characteristics, such as tumor burden and EMD status.

### 2.2 Potential factors contributing to CAR-T therapy failure

The mechanisms underlying the failure of CAR-T therapy in treating R/R MM are not yet fully elucidated, with various factors potentially contributing to treatment failure. These factors include the downregulation or loss of tumor cell target antigen expression, T-cell exhaustion, and the tumor immune microenvironment. Baseline heterozygous deletion of BCMA has been reported as a possible contributor to BCMA loss following immunotherapy, which could serve as a potential mechanism for immune escape (Da Via et al., 2021). In patients who received commercial anti-BCMA CAR-T cell therapy, the median BCMA expression decreased from 670 (interquartile range [IQR] 380–850) mol/cell prior to treatment to 390 (IQR 290–490) mol/cell post-treatment (n = 15, p = 0.011). *In vitro* studies have shown that as antigen levels decline, the tumor-lytic and cytokine production functions of CAR-T cells are compromised, and when antigen levels fall below 50 mol/cell, CAR-T cell functionality is lost (Perica et al., 2023). Beyond BCMA, a reduction or loss of GPRC5D expression has also been observed in patients who experienced disease progression after achieving remission with anti-GPRC5D CAR-T therapy (Mailankody et al., 2022; Zhang M. et al., 2023).

The expansion and persistence of CAR-T cells are closely linked to the efficacy of CAR-T therapy. Studies indicate that circulating CAR-T cell expansion exceeding 180/mm^3^ following ide-cel infusion is associated with prolonged PFS (Caillot et al., 2024). In patients with sustained best responses after cilta-cel infusion, CAR-T cell persistence was notably longer (median duration of 535.3 days–261.6 days, p = 0.01) (Xu et al., 2024). However, the human anti-mouse antibody (HAMA) response, induced by murine-derived scFv, has been shown to diminish CAR-T cell persistence (Lam et al., 2020). The tumor microenvironment in MM further influences T-cell functionality, with CD8^+^ T-cells expressing several molecules associated with T-cell exhaustion (PD-1, CTLA-4, 2B4, CD160) and T-cell senescence (CD57, loss of CD28), a phenotype correlated with reduced cell proliferation and impaired function (Zelle-Rieser et al., 2016). Moreover, high CAR expression levels are linked to increased signaling and an exhaustion phenotype, characterized by the expression of multiple inhibitory receptors, including PD-1, CTLA-4, LAG3, TIM3, and TIGIT, which is associated with diminished responses and poorer long-term PFS outcomes (Rodriguez-Marquez et al., 2022).

In recent years, the mechanisms underlying the influence of host immune status and microenvironment on CAR-T therapy responses have become increasingly well-defined. Fischer et al. conducted longitudinal flow cytometry analysis of peripheral blood mononuclear cell (PBMC) samples from 27 patients with relapsed/refractory multiple myeloma (RRMM) treated with ide-cel, revealing the critical impact of host T-cell subset distribution on therapeutic efficacy (Fischer et al., 2024). The study found that patients with a higher baseline proportion of CD8^+^ T-cells and a lower proportion of regulatory T-cells (T_regs_) were more likely to achieve remission, suggesting that the initial immune status indirectly determines treatment outcomes by modulating CAR-T expansion efficiency. Notably, patients with incomplete lymphodepletion retained CD8^+^ T-cells that potentially competed with CAR-T cells, thereby inhibiting their expansion and leading to treatment failure. Additionally, CAR-T infusion resulted in a significant increase in PD-1 expression in responders, whereas non-responders exhibited persistently high baseline PD-1 levels, indicating that a pre-existing exhausted immune environment in the peripheral blood may directly limit CAR-T functionality.

Furthermore, Rade et al. (2024) validated these findings with spatial resolution by comparing the immune microenvironment of bone marrow and PBMCs using single-cell RNA sequencing. Their study demonstrated that during leukapheresis, non-responders already exhibited pronounced immunosuppressive microenvironment characteristics: CD39^+^ monocytes suppressed local immunity via the adenosine signaling pathway, accompanied by impaired CD8^+^ T-cell and NK-cell function. Such finding aligns with Fischer’s observations on the significance of host T-cell subsets, suggesting that monocyte-mediated immunosuppression may contribute to treatment failure through multiple mechanisms. Additionally, single-cell sequencing revealed phenotypic heterogeneity within CAR-T cells: high-expansion clones exhibited cytotoxic activity but upregulated exhaustion markers such as PD-1 and CD38, whereas low-expansion clones lacked these characteristics. Such phenomenon is consistent with Fischer’s findings on PD-1 dynamics, implying that CAR-T exhaustion may have a dual biological significance: moderate activation-induced exhaustion could serve as an efficacy marker, while baseline exhaustion may indicate functional impairment.

Collectively, it is notable to highlight the pivotal role of the tumor-localized microenvironment. CAR-T cells in the bone marrow exhibited a more activated phenotype (e.g., CD69^+^, CD15^+^) than those in peripheral blood, and tumor cells with high BCMA expression were more effectively eliminated, underscoring the bone marrow as the primary site of action. Considering the critical role of lymphodepletion observed (Fischer et al., 2024), it is plausible that residual CD39^+^ monocytes in the bone marrow may suppress CAR-T function through metabolic regulation (e.g., adenosine accumulation). These findings provide new insights into combination strategies aimed at reducing CAR-T treatment failure and improving the efficacy of salvage therapy. Immunomodulatory agents targeting CD39/PIM kinases may counteract monocyte-mediated immunosuppression, while PD-1/PD-L1 inhibitors could potentially mitigate CAR-T exhaustion. For instance, preclinical studies have shown that γ-secretase inhibitors enhance CAR-T recognition efficiency by preventing BCMA shedding (García-Guerrero et al., 2022), and CD38/BCMA bispecific antibodies can synergistically overcome antigen escape. By leveraging these mechanisms, CAR-T cell persistence, recognition efficiency, and cytotoxicity could be significantly enhanced.

### 2.3 Salvage therapies

The failure of CAR-T cell therapy in relapsed/refractory multiple myeloma (R/R MM) presents a critical challenge, particularly as these therapies are often administered in late-stage disease where patients have developed resistance to conventional treatments. The median overall survival (OS) for patients with triple-refractory R/R MM is approximately 9 months, and even shorter for those with penta-refractory disease, highlighting the urgency for effective salvage strategies (Gandhi et al., 2019). Following relapse post-CAR-T therapy, the limited efficacy of subsequent treatments underscores the need for innovative approaches (Van Oekelen et al., 2023; Chinese Medical Doctor Association and Chinese Society of Hematology, 2022).

Meanwhile, As research on the underlying mechanisms of CAR-T therapy failure continues to advance, clinical salvage treatment strategies also need to evolve accordingly. It is essential to identify the primary causes of CAR-T failure, such as antigen escape (e.g., downregulation of BCMA/GPRC5D expression), T-cell dysfunction (e.g., exhaustion or insufficient expansion), and immunosuppressive microenvironments (e.g., enrichment of Tregs or MDSCs). By employing targeted biopsies (to assess antigen expression), flow cytometry (to analyze CAR-T exhaustion markers), and single-cell sequencing (to characterize the tumor microenvironment), a more precise classification of failure mechanisms can be achieved, guiding treatment selection. For example, patients with BCMA loss may require alternative targets, such as GPRC5D-directed CAR-T therapy, whereas those with T-cell exhaustion might benefit from a combination of PD-1 inhibitors and BCMA bispecific antibodies to restore T-cell activity.

Here, we outline several treatment options based on existing clinical cases, providing a reference for medical professionals and clinicians in selecting appropriate therapeutic strategies.

#### 2.3.1 Secondary CAR-T cell therapy

Secondary CAR-T therapy, whether repeating anti-BCMA CAR-T or switching to alternative targets like GPRC5D, remains a viable option for salvage therapy. However, the results from re-administering anti-BCMA CAR-T vary (Subramanian et al., 2023). While some patients respond positively (Brudno et al., 2018), the benefits tend to decrease for those previously treated with BCMA-targeted therapies (Guoxing et al., 2022). In contrast, GPRC5D-targeted CAR-T cells have shown promise in phase I trials (Zhang M. et al., 2023), achieving durable responses even after anti-BCMA therapy failure (Huang et al., 2024; Mailankody et al., 2022; Bal et al., 2023). Although the responses are likely reduced, compared to BCMA-naive patients, GPRC5D CAR-T cells continue to be a viable salvage option. Meanwhile, dual-target CAR-T cells, aiming to overcome single-target limitations, have also gained attention as the potential choice of salvage therapy. Approaches like bispecific CS1-BCMA CAR-T have demonstrated strong responses (Li et al., 2024a), while a phase I/II trial of BCMA/CD19 CAR-T achieved an 80% ORR in relapsed patients’ post-commercial CAR-T therapy (Shi et al., 2024). Moreover, universal CAR-T (UCAR-T) cells, derived from healthy donors, offer the advantage of immediate availability, enhanced T-cell targeting, and reduced manufacturing costs (Li et al., 2024b; Metelo et al., 2022). Despite the risk of mismatches causing GVHD, a phase I study showed a 60% ORR with no GVHD, though some patients experienced grade 2 CRS and ICANS (Dholaria et al., 2024).

Moreover, some novel clinical observations from CD19 CAR-T therapy provide insights into optimizing sequential CAR-T strategies. For instance, patients with relapsed B-cell malignancies who failed murine CD19 CAR-T therapy achieved durable remissions after receiving humanized CD19 CAR-T cells, likely due to reduced immunogenicity and improved persistence (An et al., 2022). Translating such experience to multiple myeloma, sequential use of BCMA CAR-T cells with distinct antigen-binding domains may help counteract antigen escape and anti-CAR immune responses. Clinical data indicate that retreatment with BCMA-directed therapies, including bispecific antibodies and alternative CAR-T constructs, can elicit responses, though durability remains a challenge (Reyes et al., 2024). Notably, patients who failed prior BCMA CAR-T may still respond to a second BCMA-directed approach, particularly with fully humanized constructs (Fu et al., 2024).

#### 2.3.2 Bispecific antibodies

Teclistamab, Talquetamab, Elranatamab, and Cevostamab are FDA-approved or in clinical evaluation as bispecific antibodies for R/R MM. These BsAbs, targeting BCMA, GPRC5D, and FcRH5 through CD3, engage T-cells to target malignant cells, offering a safer and more effective post-CAR-T therapy option (Moreau et al., 2022; Chari et al., 2022; Lesokhin et al., 2023). In their phase II trials, the prior ORRs for Teclistamab, Talquetamab, Elranatamab, and Cevostamab were 63% (Grajales-Cruz et al., 2023), 65.7% (Jakubowiak et al., 2023), 58.3% (Varshavsky-Yanovsky et al., 2023), and 54.5% (Trudel et al., 2021), respectively. With Teclistamab and Talquetamab indicated stronger responses, their safety profiles, such as grade 3 CRS (0% for Teclistamab, 5% for Talquetamab) and ICANS, were also manageable (Jakubowiak et al., 2023; Schinke et al., 2023). Comparatively, Talquetamab exhibited stronger T-cell activation in patients post-CAR-T therapy, achieving better PFS (Jakubowiak et al., 2023) and DOR (Mouhieddine et al., 2023) than those treated with BsAbs first. Similarly, Elranatamab presented favorable outcomes, particularly in African American patients, with a median overall survival (OS) of 21.2 months post-CAR-T therapy (Varshavsky-Yanovsky et al., 2023). Cevostamab, meanwhile, demonstrated notable efficacy, with ORRs ranging from 44.4% to 50%, depending on prior treatments, utilizing FcRH5 targeting to minimize severe CRS and ICANS (Trudel et al., 2021).

Together, these data indicate that BsAbs are promising options in the salvage therapy landscape, especially when used sequentially after CAR-T treatment. However, optimizing their use in combination and sequencing strategies requires further research to maximize patient outcomes.

#### 2.3.3 Novel monoclonal antibodies (PD-1/PD-L1)

The immunosuppressive effects of the tumor microenvironment are a key factor contributing to the failure of CAR-T cell therapy. PD-1/PD-L1 monoclonal antibodies have the potential to alleviate this immunosuppression, thereby enhancing T-cell activity. Currently, researchers are investigating the clinical efficacy of Pembrolizumab (Keytruda) as a monotherapy or in combination therapies for MM patients who have not responded to anti-BCMA CAR-T therapy (Chung et al., 2022). Studies have demonstrated that combining the PD-1 inhibitor Pembrolizumab with the anti-SLAMF7 monoclonal antibody Elotuzumab can induce re-expansion of CAR-T cells and enhance anti-myeloma effects. However, the best therapeutic outcome observed in this setting was a minimal response (MR) (Bernabei et al., 2018). Similarly, the use of another PD-1 antibody, Nivolumab, in R/R MM patients who relapsed after CAR-T therapy resulted in an ORR of only 18% (2/11) (Banerjee et al., 2024). Thus, the effectiveness of PD-1/PD-L1 monoclonal antibodies as a salvage therapy for R/R MM after CAR-T cell therapy failure remains an area that requires further investigation and optimization.

#### 2.3.4 Antibody-drug conjugates (ADCs)

Antibody-drug conjugates (ADCs) like Blenrep (Belantamab mafodotin) are designed to target cancer cells with an antibody linked to a cytotoxic payload. Approved by the FDA in August 2020 for R/R MM patients who had undergone at least four prior therapies, Blenrep was withdrawn from the U.S. market in November 2022 due to failing to meet the primary endpoint of progression-free survival (PFS) in the phase III DREAMM-3 trial.

In clinical evaluations, Blenrep achieved an overall response rate (ORR) of 33%. However, among seven patients previously treated with anti-BCMA CAR-T therapy, none responded to Blenrep; two had stable disease and five progressed (Vaxman et al., 2021). Meanwhile, in the KarMMa phase II trial, a patient resistant to a second CAR-T therapy achieved a very good partial response (VGPR) after three doses of Blenrep, with the response lasting 5 months (Gazeau et al., 2021). Additionally, a single-center retrospective study (Subramanian et al., 2023), found that among 30 R/R MM patients who progressed after anti-BCMA CAR-T therapy, those receiving Blenrep had an ORR of only 25% and a median PFS of 1.8 months. These results highlight the need for further research to optimize ADC therapies in this setting.

#### 2.3.5 Other treatments

For R/R MM patients relapsing after CAR-T therapy, traditional therapeutic methods remain alternatives including proteasome inhibitors, immunomodulatory agents, and nuclear export inhibitors. The LEGEND-2 study (Liu et al., 2024) showed that proteasome inhibitor-based therapies were highly effective in these patients, achieving an overall response rate (ORR) of 50%, significantly higher than re-administered BCMA CAR-T (30%) or combination chemotherapy (0%). This suggests that CAR-T therapy may re-sensitize myeloma cells to previously used drugs.

Ferreri et al. (2023), evaluated 30 R/R MM patients after anti-BCMA CAR-T therapy failure, exploring various salvage therapies such as repeat BCMA CAR-T therapy, stem cell transplantation, bispecific antibodies, and anti-CD38 monoclonal antibodies. The highest efficacy was observed with repeat BCMA CAR-T and stem cell transplantation (Subramanian et al., 2023). Additionally, a case study by Agte et al. showed that a patient with BRAF V600E-mutated MM responded well to a BRAF inhibitor-based regimen, reducing free λ light chains by 80% (Agte et al., 2021). Moreover, another patient with extramedullary disease (EMD) achieved a stringent complete response (sCR) following haploidentical hematopoietic stem cell transplantation (HSCT) (Qian et al., 2021). These findings emphasize the importance of personalized treatment strategies and further research to optimize salvage therapy options for patients relapsing after CAR-T therapy.

Moreover, radiation therapy (RT) has recently emerged as a salvage strategy for localized disease control in relapsed/refractory MM, particularly for patients with extramedullary plasmacytomas or bone lesions refractory to systemic therapies. A study have demonstrated that focal RT can synergize with CAR-T therapy by reducing tumor bulk and modulating the immunosuppressive microenvironment, potentially enhancing CAR-T cell infiltration and activity (Ababneh et al., 2023). For instance, among patients with oligoprogressive disease post-BCMA CAR-T failure, RT to isolated lesions may delay systemic progression and provide a bridge to subsequent therapies. However, further investigation is needed to optimize RT timing, dose, and combination approaches with immunotherapies.

### 2.4 Recommended strategies

The approval and commercialization of Ide-cel have ushered in a new era of immune cell therapy towards R/R MM, with the subsequent approval of Cilta-cel for second-line treatment further emphasizing the pivotal role of CAR-T cell therapy in this context. However, this advancement brings forth critical research challenges, such as the need to extend progression-free survival (PFS) and overall survival (OS) associated with CAR-T therapy, as well as determining the most effective salvage treatment strategies following disease relapse or progression. Based on the findings from relevant clinical studies, we propose the following recommendations.

#### 2.4.1 Exploring CAR-T cells with different targets and origins

For R/R MM patients after CAR-T therapy, it is recommended to initially assess the expression of antigens such as BCMA and GPRC5D on the surface of MM cells and select CAR-T cell therapy targeting the corresponding antigens. Currently, CAR-T cells targeting BCMA have been approved for marketing, while those targeting other antigens such as GPRC5D are undergoing clinical trials. Treatments based on anti-GPRC5D CAR-T cells (Mailankody et al., 2022; Zhang M. et al., 2023; Bal et al., 2023), anti-BCMA CAR-T (Subramanian et al., 2023) or dual-target CAR-T cells (Shi et al., 2024) have been shown to be effective for patients who have failed previous anti-BCMA CAR-T cell therapy, and we recommend using CAR-T cells with different targets from those previously used as the first line of therapy. Additionally, considering that different sources and constructs of scFv can affect the efficacy of CAR-T cell therapy (Lin et al., 2019; Berdeja et al., 2021), it is also possible to choose humanized or fully human CAR-T as salvage therapy following the progression of CAR-T treatment.

#### 2.4.2 Rapid-manufactured CAR-T

During the conventional autologous CAR-T cell manufacturing process, patients with a high tumor burden often require bridging therapy to manage disease progression. However, the rapid advancement of the disease in some patients can preclude the administration of CAR-T cell therapy, potentially leading to mortality before the therapy can be administered (Ahmed et al., 2023). Moreover, bridging therapy can also affect subsequent efficacy, such as longer median hospital stay, shorter median progression-free survival (PFS) (8.1 months–11.5 months, p = 0.03), and shorter median overall survival (OS) (13.8 months to not reached, p = 0.002) (Afrough et al., 2023). Rapid CAR-T cell manufacturing processes, by shortening production time, yield T-cells with lower differentiation, requiring fewer cell doses, reducing production costs, and enhancing *in vivo* expansion of CAR-T cells, thereby potentially offering significant advantages in the treatment of R/R MM. In addition, the advantages of rapid manufacturing also include reducing bridging therapy and mitigating adverse events following bridging therapy. Therefore, rapidly manufactured CAR-T cells are a very promising type of CAR-T cell. CT071 is manufactured using an expedited CARcelerate™ platform which shortens the manufacturing process to around 30 h, and achieved an ORR of 94.1% in R/R MM patients (Du et al., 2024)Among them, four patients with previous exposure to BCMA or BCMA/CD19 CAR-T cell therapy achieved responses (2 sCR and 2 PR). Novartis™ has developed an anti-BCMA CAR-T cell product, PHE885, using its T-charge platform for rapid manufacturing (less than 2 days), which achieved an ORR of 98% in R/R MM patients who had undergone a median of four prior lines of therapy (Sperling et al., 2023). The incidence of CRS was 96%, with 11% experiencing grade 3 C RS, while ICANS occurred in 22%, with 7% experiencing grade 3 ICANS. Additionally, other companies such as Gracell Biotechnologies (Du et al., 2023), BMS (Costa et al., 2022), and IASO Bio (Liu et al., 2022) are also developing rapidly manufactured CAR-T cells for the treatment of R/R MM, featuring a desirable landscape of the application of rapid-manufactured CAR-T towards the failure of post CAR-T therapies.

#### 2.4.3 Sequential selection of T-Cell redirecting therapies

The optimal sequencing of T-cell redirecting therapies in relapsed/refractory multiple myeloma (R/R MM) is a multifaceted challenge that must balance treatment durability, antigen targeting, and patient-specific factors. According to the latest IMWG consensus (Costa et al., 2025), some novel recommendations should guide sequencing strategies to maximize efficacy, preserve T-cell functionality, and overcome resistance mechanisms.

Currently, evidence supports that prioritizing CAR-T therapy over bispecific antibodies (BsAbs) when feasible. CAR-T cells have been shown to induce deep and durable responses that provide extended treatment-free intervals, thereby allowing immune recovery and reducing the selective pressure on tumor clones (Reyes et al., 2024). In contrast, prolonged BsAb exposure may lead to T-cell exhaustion, potentially compromising the success of subsequent immunotherapies. Meanwhile, for patients who relapse after receiving BCMA-targeted CAR-T therapy, switching to an alternative target, such as GPRC5D, is a strongly recommended strategy according to the IMWG consensus (Costa et al., 2025). Clinical trial data such as POLARIS (Huang et al., 2024), which applied anti-GPRC5D CAR-T, reported a 100% overall response rate (ORR) for GPRC5D CAR-T in patients who had previously failed BCMA CAR-T therapy, proved that such strategy is a considerable choice, while other choices such as anti-GPRC5D BsAb also might generate remission. Moreover, dual-target CAR-T constructs (e.g., combining BCMA with GPRC5D or CD19) have demonstrated ORRs between 80% and 93% in early-phase studies (Li et al., 2024a; Shi et al., 2024), suggesting that such application also might effectively mitigating the risk of antigen escape. In contrast, retreatment with the same BCMA CAR-T product generally results in response rates below 30%, likely due to antigen loss and T-cell dysfunction (Hu et al., 2023). Notably, BsAbs depend on the patient’s own T-cells, which raises concerns about potential T-cell dysfunction (Choi and Kang, 2022). Due to differences in toxicity profiles, expected response rates, and administration methods, CAR-T cell therapy is generally more suitable for younger patients, those with aggressive or relapsed disease, and those with a high tumor burden (Kegyes et al., 2022).

The sequence of therapy also critically influences subsequent treatment efficacy. Clinical data support this approach. In a real-world study, prior BCMA-targeted therapy reduced median PFS for ide-cel from 9.0 to 6.2 months (Dima et al., 2024), and in CARTITUDE-2, patients previously treated with BCMA-targeted agents had a lower ORR (60%) and median PFS (9.1 months) compared to BCMA-naïve patients (Cohen et al., 2023). Conversely, BsAb therapy following CAR-T remains effective, with talquetamab achieving a 72.9% ORR in prior CAR-T recipients versus 56.5% in those previously treated with BsAbs (Jakubowiak et al., 2023). Notably, BsAbs were less effective when used immediately after another BsAb, suggesting cumulative T-cell exhaustion (ORR, 28.6%–66.7%). Given that CAR-T cell therapy can induce profound and durable responses with extended treatment-free intervals, thereby reducing the risk of T-cell exhaustion and the selective pressure from continuous therapy (Reyes et al., 2024), it is recommended that CAR-T cell therapy be prioritized in the treatment course of MM, with BsAb therapy considered upon disease progression after CAR-T treatment.

Meanwhile, it is important to note that current clinical data on sequential treatment with CAR-T and bispecific antibodies primarily come from small-sample, single-arm studies. The heterogeneity of study populations, such as the proportion of penta-refractory patients and prior exposure to BCMA-targeted therapies, may affect the generalizability of conclusions. For instance, in the MonumenTAL-1 study, 30% of enrolled patients were penta-refractory (Schinke et al., 2023), whereas this proportion was only 15% in the CARTITUDE-1 trial (Berdeja et al., 2021). This highlights the need for personalized sequential selection strategies based on prior therapy, tumor burden, and immune fitness.

#### 2.4.4 Combination therapy

Combining CAR-T therapy with other drugs can enhance the persistence of CAR-T cells *in vivo*, improve BCMA expression, and reduce T-cell exhaustion. Such drugs include immunomodulatory agents, γ-secretase inhibitors, and CRBN E3 ligase modulators.

Lenalidomide is a commonly used immunomodulatory drug (IMiD) that was approved by the FDA for the treatment of MM in June 2006. Preclinical studies have shown that lenalidomide can provide a co-stimulatory effect on CS1 CAR-T cells, enhancing their antitumor activity and persistence by improving CS1 CAR-T cell cytotoxicity, memory maintenance, Th1 cytokine production, and immunological synapse formation (Wang et al., 2018). Lenalidomide can regulate the ICOSL/ICOS pathway, Th1/Th2 pathway, increase CAR-T cell secretion of cytokines such as IFN-γ, IL-2, and TNF-α, and enhance CAR-T cell cytotoxicity (Works et al., 2019). Researchers reported a case of an R/R MM patient who relapsed after multiple chemotherapies, autologous hematopoietic stem cell transplantation, murine CAR-T cell therapy, and human CAR-T cell therapy. After lymphodepletion with fludarabine and cyclophosphamide, the patient was given lenalidomide on day −1, followed by human CAR-T cell infusion on day 0, achieving VGPR within 14 days and maintaining it for over 8 months (Zhao et al., 2022).

BCMA is cleaved from the surface of MM cells by γ-secretase. Preclinical studies have indicated that inhibiting γ-secretase can increase BCMA expression in MM cells, thereby enhancing the efficacy of CAR-T cell therapy (Pont et al., 2019). A phase I clinical study evaluated the safety of crenigacestat, a γ-secretase inhibitor, in combination with anti-BCMA CAR-T cells for the treatment of R/R MM patients. The results showed that the γ-secretase inhibitor could block BCMA shedding, overcoming antigen downregulation and thereby improving CAR-T cell efficacy (Cowan et al., 2023).

*In vitro* studies on the CRBN E3 ligase modulator iberdomide have demonstrated that iberdomide can significantly reduce the transcription factors Ikaros and Aiolos in CAR-T cells, promote the persistence and proliferation of IL-2-starved cells, significantly increase the expression of Ki67 in CAR-T cells, enhance cell viability, upregulate activation markers such as HLADR and CD69, downregulate TIGIT expression, and increase the expression of IL-2, IL-17a, and TNF-α. Therefore, iberdomide shows promise in assisting CAR-T cell therapy by overcoming CAR-T cell resistance and enhancing its efficacy (Aleman et al., 2023).

Based on these findings, combining CAR-T cell therapy with related drugs may improve the efficacy of CAR-T treatment from multiple aspects, potentially extending PFS and OS in patients.

#### 2.4.5 Maintenance therapy

A survey conducted by the International Myeloma Society revealed that 30% of the clinicians surveyed administer maintenance therapy after CAR-T cell treatment, primarily to sustain the level of remission achieved or to deepen the response further (Hassan et al., 2023). However, a survey by the American Society for Transplantation and Cellular Therapy (ASTCT) Practice Guidelines Committee found that 86% of clinicians would not consider maintenance therapy in the absence of biochemical or clinical evidence of myeloma, with only 18% considering it for patients who achieved a partial response (PR) after CAR-T cell therapy but did not convert to a complete response (CR) after several months of follow-up (Hashmi et al., 2024b). Therefore, it is recommended to identify risk factors before initiating CAR-T cell therapy, assess which patients might benefit from maintenance therapy, and determine the appropriate regimens. Continuous monitoring of disease status post-CAR-T cell therapy is crucial, with maintenance therapy being initiated at the optimal time to prolong progression-free survival (PFS) and overall survival (OS).

In a study by Li et al. (2024a), the use of bispecific CS1-BCMA CAR-T cells in treating R/R MM patients resulted in an overall response rate (ORR) of 81% (13/16). Within the study, three patients (Patients 10, 11, and 15) received lenalidomide as maintenance therapy (10 mg/day, 21 days of a 28-day cycle) following CAR-T cell treatment. Patients 10 and 11 achieved deeper responses; however, Patients 10 and 15 eventually experienced relapse or progression, while Patient 11 continued to maintain a stringent complete response (sCR). Another case involved an R/R MM patient who developed secondary plasma cell leukemia and was treated with anti-BCMA CAR-T cells, achieving a sustained sCR for 9 months. This patient then received intermittent venetoclax treatment at 10 mg/day, maintaining the response for an additional 7 months (Deng et al., 2022). In the phase II CARTITUDE-2 clinical study (Arnulf et al., 2024) evaluating Cilta-cel for newly diagnosed MM patients with suboptimal response to first-line autologous stem cell transplantation, 17 patients were assessed. Among all the patients, five received Cilta-cel alone, while 12 began continuous lenalidomide maintenance therapy (for up to 2 years) 21 days after Cilta-cel infusion. The results showed a general ORR of 94%, with both the 18-month PFS rate (as assessed by investigators) and OS rate at 94%, suggesting that a single infusion of Cilta-cel combined with lenalidomide maintenance therapy can produce a durable, deep response. These findings indicate that incorporating lenalidomide as maintenance therapy after CAR-T cell treatment may be an effective strategy to address CAR-T cell therapy failure.

## 3 Conclusion

Although CAR-T cell therapy has significantly improved the efficacy of treatment for R/R MM, factors such as downregulation or loss of tumor antigen expression, T-cell exhaustion, and the tumor immune microenvironment can lead to CAR-T therapy failure. Various salvage therapies, including repeat CAR-T cell treatments and sequential selections, have been employed in clinical practice with positive outcomes. It is recommended to develop individualized treatment plans based on the patient’s specific condition, selecting the most appropriate therapies to help R/R MM patients who have experienced CAR-T failure achieve prolonged remission, while integrating miltiple treatment strategies, including RT, may further enhance disease control and facilitate transition to subsequent systemic treatments.

At the same time, while the reviewed studies provide valuable insights into salvage therapies post-CAR-T failure, several limitations must be acknowledged. First, many trials have small sample sizes, which may limit the generalizability of findings. Second, the heterogeneity of patient populations, such as differences in prior treatment lines, extramedullary disease prevalence, and tumor burden, complicates direct comparisons across studies. Third, the lack of standardized criteria for defining CAR-T failure and selecting salvage therapies introduces variability in clinical outcomes. Future research should prioritize larger, multicenter studies with stratified patient cohorts to address these limitations and establish evidence-based guidelines for salvage therapy. Future research should focus on developing a stratified treatment framework based on failure mechanisms. For instance, dual-target CAR-T therapies (e.g., BCMA/GPRC5D) could be designed for patients with antigen escape, while immunosuppresive target inhibitors could be explored as combination therapies for patients with a predominantly immunosuppressive microenvironment. Additionally, the potential of epigenetic modulators, which are about to enhance efficacy in patients with T-cell exhaustion should be evaluated. By integrating multi-omics dynamic monitoring with mechanism-driven interventions, the precision of salvage therapies can be significantly improved. Besides, most of the current literature consists of small retrospective case series, highlighting the need for larger studies in the future to establish standardized salvage treatment protocols for R/R MM patients following CAR-T therapy failure.
